# Camel Bite Injury to the Face in an Adult Patient: Skin Closure Controversy

**DOI:** 10.7759/cureus.19765

**Published:** 2021-11-20

**Authors:** Hany A Zaki, Eman E Shaban, Ahmed E Shaban, Haitham Hodhod, Amr Elmoheen

**Affiliations:** 1 Emergency Medicine, Hamad Medical Corporation, Doha, QAT; 2 Cardiology, Aljufairi Diagnostic and Therapeutic Hospital, Doha, QAT; 3 Internal Medicine, Mansoura General Hospital, Mansoura, EGY; 4 Emergency Medicine, King's Mill Hospital, Mansfield, GBR

**Keywords:** wound infection, postoperative wound infection, skin closure, face injury, superficial cervical plexus block, primary fascial closure, soft tissue injuries, mammalian bite, animal bite management, camel bite

## Abstract

Animal bite injuries are prevalent worldwide. Camel bites, as a cause, are relatively rare. Male camels are particularly aggressive, especially during the rutting season. These injuries, when inflicted over the face, have a disfiguration effect with possible psychological repercussions to the patient. The surgical management of facial camel bite is described sporadically and remains a source of deliberation. Our paper reports the mechanism and management of facial soft tissue injury inflicted by camel bite over the face in an adult male with long-time follow-up for the patient post surgical repair without any documented complications. This case report demonstrates the complex nature of camel bite injuries over the face. Inappropriate wound management may result in long-term sequelae, which may affect the patient’s quality of life. Individuals should apply caution when dealing with camels, mainly in the rutting season. Primary skin closure, especially to the face or neck, and proper wound management will decrease the risk of permanent scars and infections.

## Introduction

Camels are domestic animals that serve as a means of transportation in desert regions and other areas where modern transportation is near impossible. It is a simple animal, very quiet, and usually very obedient to its proprietor. But then, it has been observed that the threshold of tolerance of these animals reduces drastically during their breeding season. The camel breeding season begins from December right up to March. During the breeding season, people close to them become very vulnerable to their attacks.

It is important to note that animal bite injuries vary based on geographical distribution, animal anatomy, and behavior. Human injuries due to camel bites are a rarity, and the injuries and attacks become more common during the rutting season. At this time, male camels become very aggressive [1]. Camel bites have a very complex mechanism, and this explains why it is associated with high morbidity. Injuries to the neck and head are usually frequent and severe [2]. Injuries may include skull fractures, facial wounds, cervical neurovascular injuries, and intracranial bleeding [1,2].

Most studies reporting camel bites are retrospective in nature, and not many case studies have been documented. A Saudi Arabian study reporting animal-related injuries submitted that out of 13 cases, ten were due to camels; therefore, this translates to 77%, and two of these were camel bites (15%) [3]. A Nigerian study reported that camels caused 9 out of 34 animal-related injuries (translating to 26%). Of these, two were attributed to camel bites [4]. A third study also conducted in Nigeria reported that 6 out of 9 camel-related injuries (translating to 67%) were upper limb bites [5].

Injuries caused by camels are unusual, severe, and usually maxillofacial [6,7,8]. The injury does not have a fixed pattern but usually involves the zygoma, maxilla, mandible, orbit, and nose. Over 60-70% of camel bites involve the neck and head region because these regions are easily accessible [9]. The precise incidence of camel bites is not known, and very few cases have been documented. As such, a detailed review of this subject will be of immense interest to the reader [6,7].

We report a rare case of camel bite injuries to the face of an adult patient that resulted in irregular, superficial to deep lacerations and contusions over the left external ear region, left side of the face over the parotid gland, and neck.

## Case presentation

A young adult male, previously healthy, was referred to our accident and emergency department by a primary health center, with a history of being bitten on the face by a male domestic camel while he was attempting to feed it. The camel attacked, as it was confused between him and another human who had previously harmed the camel physically. This behavior is part of a camel's vengeful nature.

The camel bite included the left side of the face, left ear, and neck. No other obvious injuries were observed nor was there a history of loss of consciousness, vomiting, or recent or current medical illness. No recent hospital admission or previous surgical intervention was reported. In addition, no history of known allergies was noted.

Clinical examination

**Figure 1:**
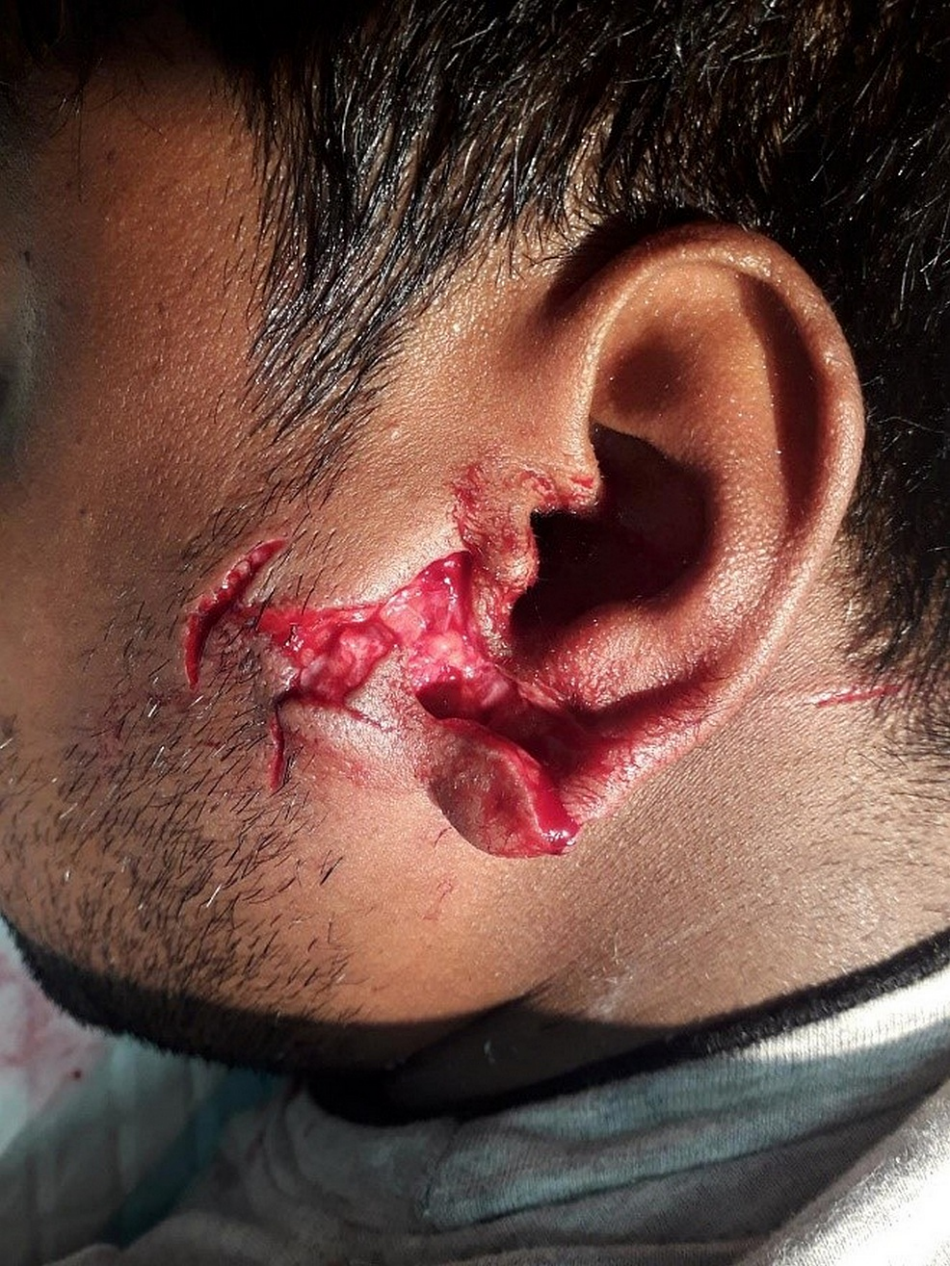
Multiple irregular, superficial to deep lacerations, and contusions over the left external ear region, left side of the face over the parotid gland, and neck.

The patient appeared to be in pain and anxiety, but not in respiratory distress, and had normal vital signs. He had irregular, superficial to deep lacerations, and contusions over the left external ear region, left side of the face over the parotid gland, and neck. There was no obvious bleeding from the ear canal, parotid gland injury, hearing affection, or pain with mastication which might indicate (temporomandibular joint [TMJ] dislocation/subluxation) (Figure 1). Neurological examination of the fifth and seventh cranial nerve was unremarkable, and the rest of the facial bones were intact. Spine, chest, abdomen, and extremities examinations did not reveal any injury.

Management and follow-up

**Figure 2:**
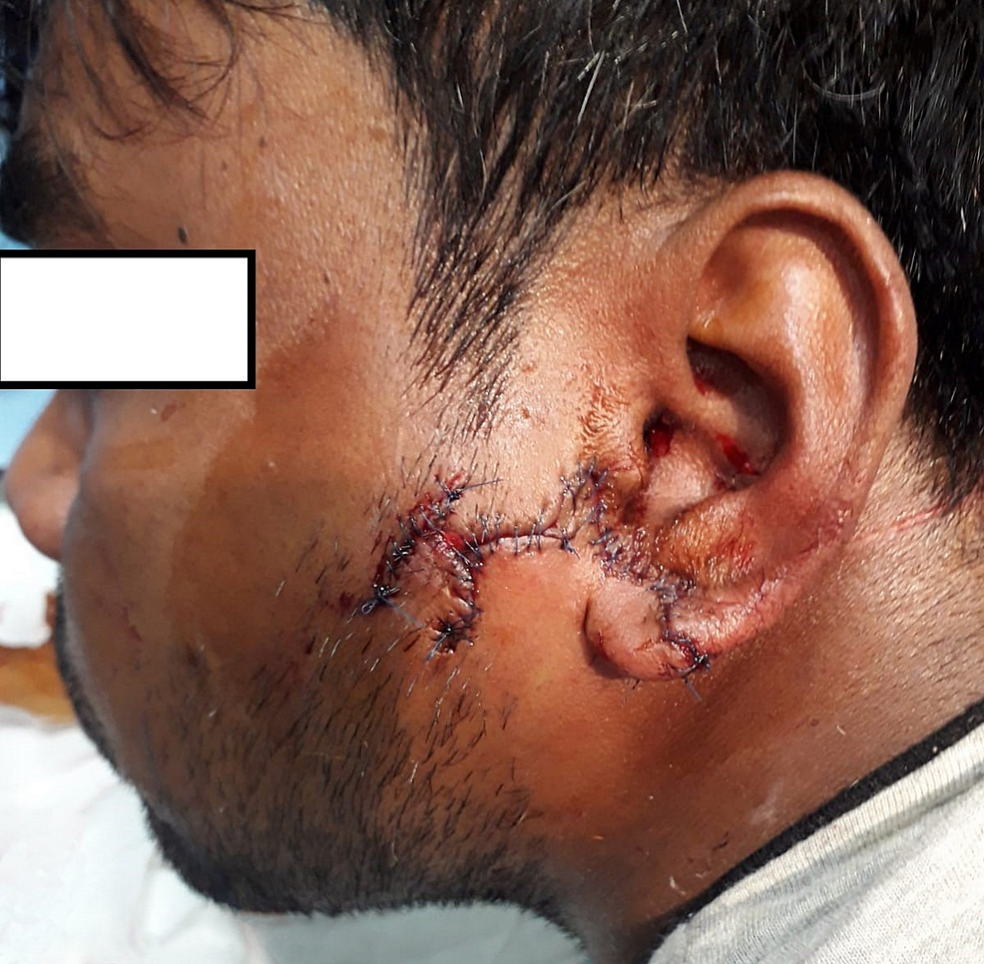
The facial and ear lacerations were closed primarily, continuous type, after judicious debridement of the devitalized tissue.

Before starting wound cleaning and debridement, we performed a wound swab that was sent for the microbiology culture and sensitivity to be a guide for the antimicrobial therapy. Prophylactically, IV antibiotics and cefazolin plus metronidazole (10 mg/kg) were administered. Passive immunization with 0.5 ml of tetanus toxoid vaccine and 1 ml of inactivated rabies virus vaccine intramuscularly at 0, 3, 7, 14, and 28 days after exposure with 20 IU/kg body weight of human rabies immunoglobulin, as per Schedule C of WHO 1997 guidelines, were administered. The wounds were thoroughly irrigated with 3% hydrogen peroxide solution, 5% povidone-iodine solution, and finally by copious isotonic saline under superficial cervical plexus block guided by ultrasound by using bupivacaine 0.25%, 10 ml. The facial and ear lacerations were closed primarily, continuous type, after judicious debridement of foreign bodies and devitalized tissue. He was subsequently discharged from the hospital in satisfactory condition with no residual functional or aesthetic disability (Figure 2).

**Figure 3:**
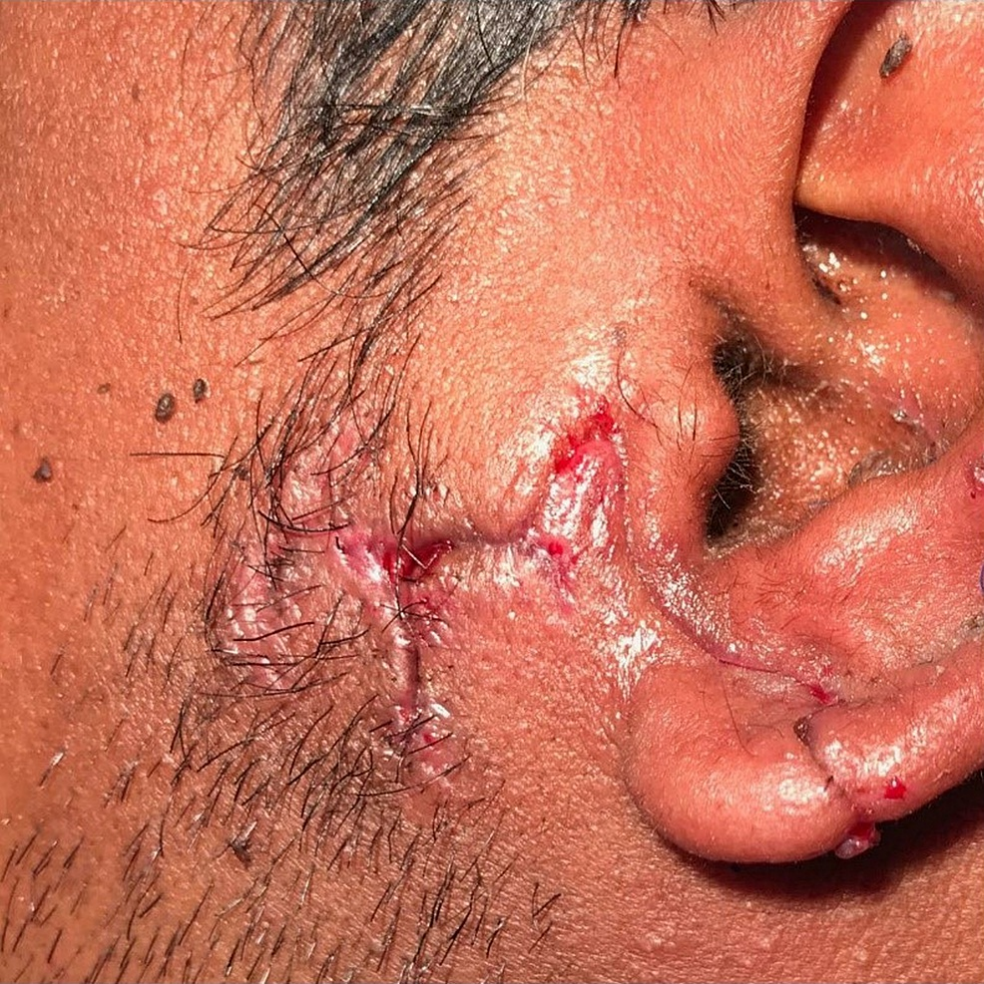
The wound showed good healing in the follow-up appointment for sutures removal 10 days post suturing.

**Figure 4:**
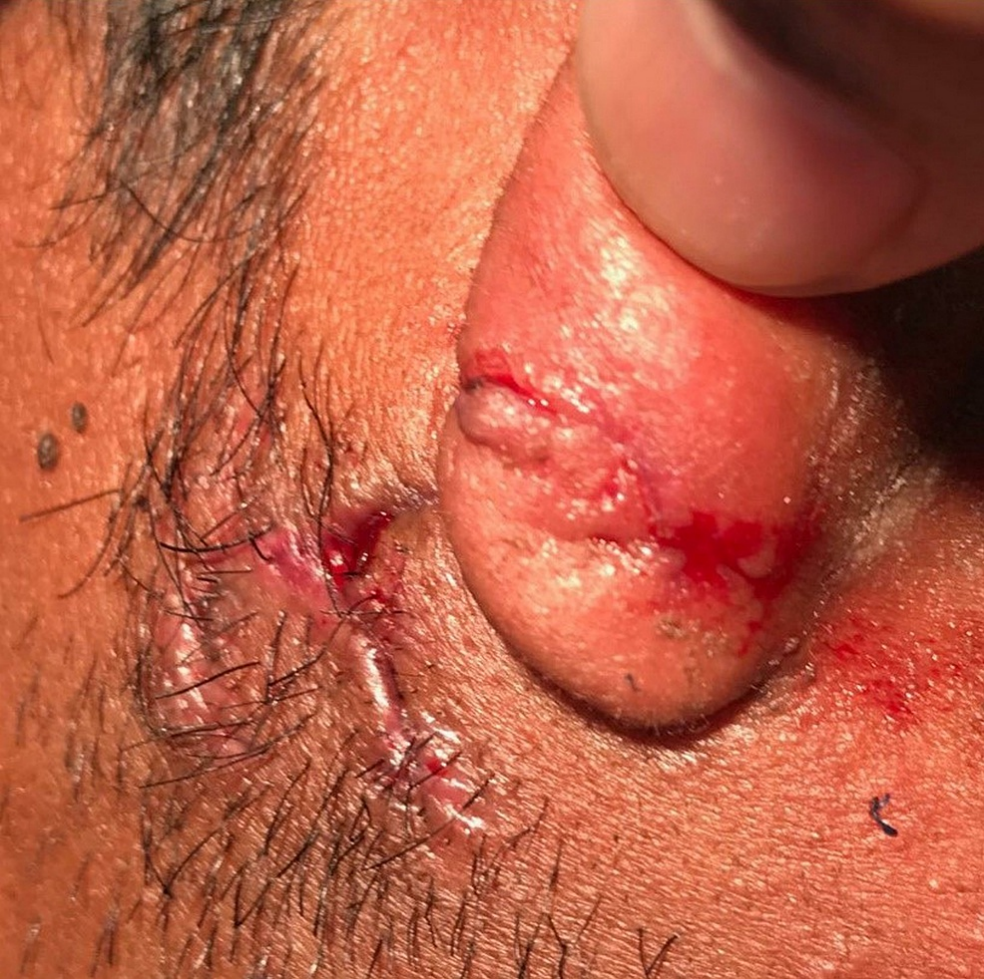
Another post-suture removal view; The wound showed good healing in the follow-up appointment for sutures removal 10 days post suturing.

Daily dressing with follow-up and clear instructions were given to the patient in case of any compromise symptoms, with a prescription on both amoxicillin/clavulanate, 1 gm orally twice daily for a total of 10 days with oral metronidazole 400 mg orally twice daily for a total of two weeks. Results of the wound culture came back 48 hours later, showing growth of Actinobacillus and Pasteurella. Both organisms were sensitive to amoxicillin and metronidazole. Based on these results, the patient was contacted and was updated about the results, and was advised to continue the previously prescribed oral antibiotics courses. The wound showed good healing in the follow-up appointment for sutures removal 10 days post suturing (Figures 3-4).

## Discussion

Camel bite injuries are rare, and the patient in our study was bitten by a male camel. Most camel bites occur in the rutting season, which begins from December and extends through March. At this time, the camels become irritable, hard to handle, and their behavior becomes highly unpredictable.

Bites from a camel can cause severe lacerations and puncture wounds. A camel's bite has the potential to injure deep body structures despite the wound's superficial appearance. Skull fractures, facial bone fractures, and brain injuries are possible complications of camel bites to the face and head [2,4,10]. Severe injuries like brain infarctions and carotid artery occlusions can result from camel bites to the neck [1]. The severe nature of camel bite injuries is attributed to its dentition, which is unique. A mature camel has 34 teeth with four very sharp and long canine teeth [11]. Canine teeth caused the deep lacerations and contusions observed in our patient.

Camel bite injuries have a very complex mechanism. This includes crushing and penetrating wounds by the camel's strong jaws and sharp teeth, as well as blunt injuries when they come, picks up, shakes, and throws its victim [1,12]. The repeated bites experienced by our patient resulted in superficial to deep lacerations over the region of the left external ear and the left side of the face over the neck and the parotid gland (Figure 1).

In the present case, injury to the parotid gland was due to the penetrating injury of the cheek along the line linking the tragus of the ear to the lip midportion [13]. Because of the close association of the parotid duct with the buccal branch of the facial nerve, trauma to the parotid duct or gland will certainly raise suspicion of injury to the facial nerve.

Surgical management of bite injuries caused by animals remains controversial among clinicians who handle such cases [14]. Treatment of each case depends on several factors, including the nature and location of the injury and the type of animal [15].

Some authorities advise against suturing of the injury due to probable contamination while also recommending rabies prophylaxis because it remains the major complication secondary to animal bite [16]. However, in the present case, we administered passive immunization with 0.5 ml of tetanus toxoid vaccine and 1 ml of inactivated rabies virus vaccine intramuscularly at 0, 3, 7, 14, and 28 days after exposure with 20 IU/kg body weight of human rabies immunoglobulin as per Schedule C of WHO 1997 guidelines. The facial and ear lacerations were closed primarily, continuous type, after judicious debridement of foreign bodies and devitalized tissue. Antibiotics were continued seven days postoperatively, after which the patient was discharged in satisfactory condition.

Management of complex injuries such as this requires several protocols and methods [17]. Tissue and fracture repairs require special focus because they are difficult (technically) [18]. Communication, for instance, increases the risk of ischemic compromise plus vascular necrosis of fragments, potentially leading to infection, nonunion, and continuity defect [19,20]. In such cases, it becomes challenging to reconstruct both hard and soft tissues. As such, it is important to adopt a multidisciplinary approach to get good results [21,22].

## Conclusions

In conclusion, our case demonstrates the complex nature of camel bite injuries over the face. Poor treatment may result in long-term sequelae, which may affect the patient’s quality of life. Individuals should apply caution when dealing with camels, mainly in the rutting season. Primary skin closure of face wounds and antibiotics prophylaxis with meticulous care may lead to a good prognosis without complication.
